# Lung Herniation Associated With Crack Cocaine Use: An Uncommon Cause of Chest Pain

**DOI:** 10.7759/cureus.21801

**Published:** 2022-02-01

**Authors:** Thales Nogueira Gomes, Mariana Camelo Pereira, Sarah C Smith, Thomas A Melgar

**Affiliations:** 1 Internal Medicine, Western Michigan University Homer Stryker M.D. School of Medicine, Kalamazoo, USA

**Keywords:** chronic obstructive pulmonary disease, crack cocaine use, intrathoracic pressure, chest pain, traumatic lung hernia, cocaine use, pulmonary herniation, lung herniation

## Abstract

Lung herniation is an uncommon clinical entity characterized by protrusion of pulmonary tissue through an area of weakness in the chest wall. We present the case of a 56-year-old man with a history of chronic obstructive pulmonary disease (COPD) and crack-cocaine use who presented to the emergency department due to left-sided lateral chest pain, as well as a two-week history of cough, shortness of breath, and wheezing. Chest imaging revealed a contusion on the left flank and intercostal widening with a left-sided pulmonary herniation between ribs 8 and 9. Cardiothoracic surgery was consulted for assessment of pulmonary herniation and recommended conservative management. His pain was managed with multimodal analgesia and the patient was deemed stable for discharge. At outpatient follow-up two weeks later, his pain was well-controlled. To our knowledge, this is the first reported case of pulmonary herniation in which crack cocaine use is implicated as a contributing cause. The outcome achieved in our case supports the use of conservative management with analgesia as a valid strategy for select patients with lung herniation.

## Introduction

Lung herniation, defined as protrusion of the organ beyond the chest wall, is an uncommon condition, with approximately 300 cases documented in the literature [1], therefore being frequently overlooked and misdiagnosed. It can present in a variety of ways, such as thoracic pain and/or visible deformity as well as dyspnea [2]. Pulmonary herniation is most commonly associated with trauma or surgery; however, it has also been reported to happen spontaneously or after severe coughing episodes [3]. Diagnosis may be suggested by physical exam findings but will ultimately be confirmed by imaging, usually CT. We present a case of pulmonary herniation caused due to blunt trauma to the left hemithorax and vigorous coughing fits.

## Case presentation

We present the case of a 56-year-old man with a history of moderate/GOLD stage B [4] chronic obstructive pulmonary disease (COPD), crack-cocaine use, current cigarette smoking, and obesity who presented to the hospital due to left-sided lateral chest wall tenderness. The patient reported that he had been having worsening pain for the previous two weeks. He showed some bruising to the chest wall, which he attributed to falling during a syncopal event associated with a coughing spell around the time when symptoms started. Additionally, the patient had noticed worsening of his chronic cough, which exacerbated the pain, as well as shortness of breath, pleuritic chest pain, and wheezing.

**Figure 1:**
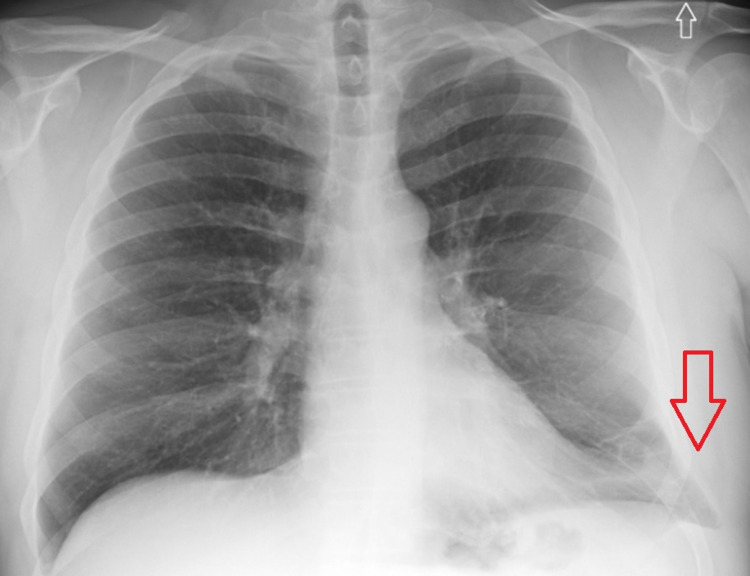
Posteroanterior chest radiography evidencing protrusion of pulmonary tissue beyond costal margins in the left lower lung aspect (red arrow).

On initial evaluation, he was found to be hypoxic with oxygen saturation of 80% and was briefly started on bi-level positive airway pressure (BiPAP). After six hours, he was successfully weaned to oxygen nasal cannula. On exam, wheezing and significant tenderness to palpation of the left anterolateral thoracic wall were noted. He also had a large area of ecchymosis on the left chest wall extending to the left flank. Labs were significant for leukocytosis (18.5 x103 leukocytes). Urine drug screen was positive for cannabinoids and cocaine. Electrocardiogram (EKG) showed normal sinus rhythm without ST-segment changes. Chest radiography was negative for any acute processes (Figure 1).

**Figure 2:**
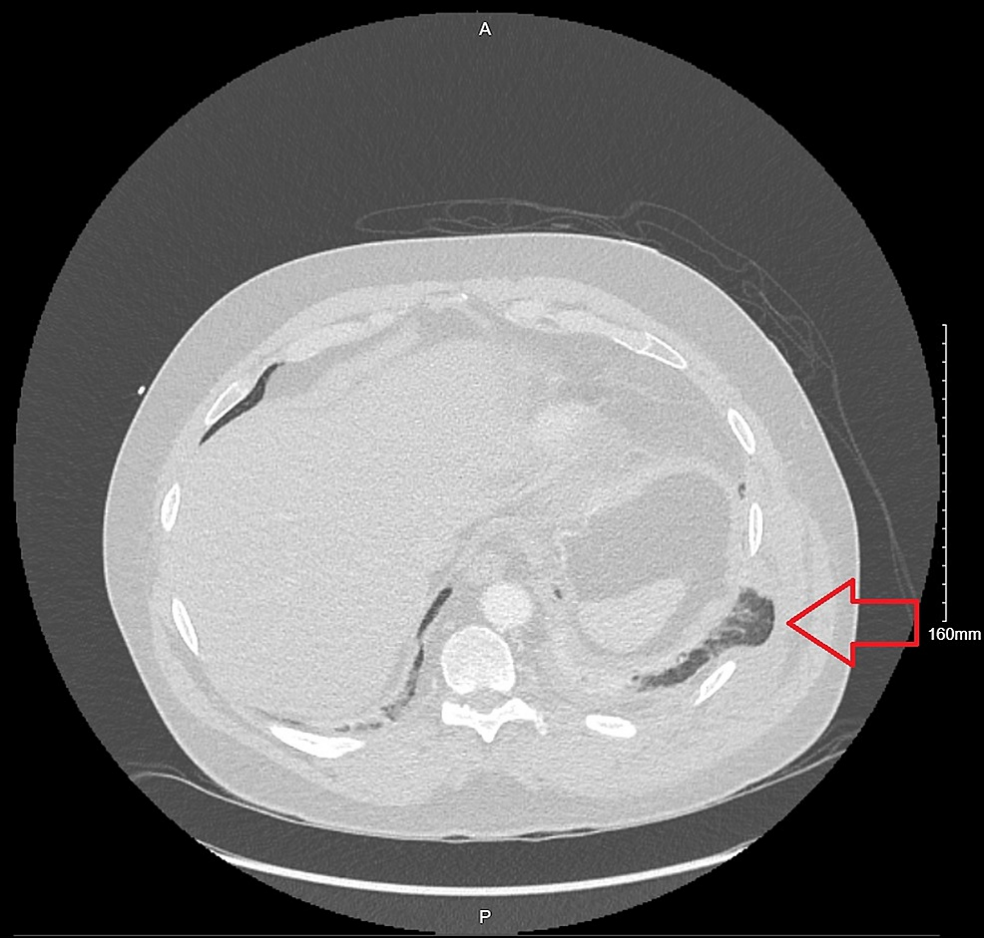
Axial view chest CT imaging showing area of intercostal widening with lung herniation on left lower thoracic region, between ribs 8 and 9 (red arrow).

**Figure 3:**
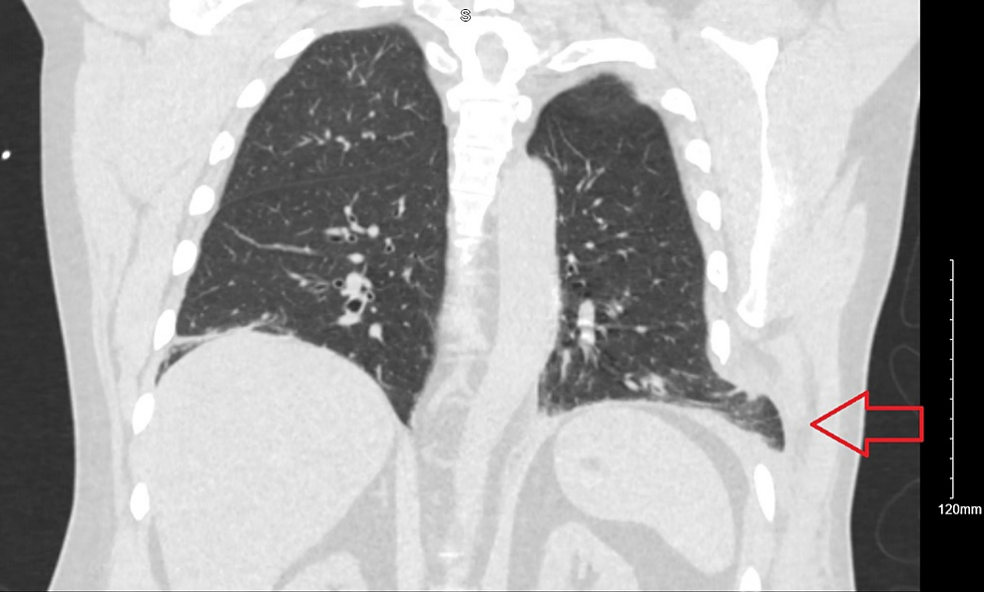
Coronal view of chest CT imaging highlighting area of lung herniation (red arrow) in left lower thoracic wall.

A CT of the chest showed bibasilar infiltrates, small left pleural effusion, and a contusion on the left flank. It also showed a left-sided pulmonary herniation between ribs 8 and 9, without any evident rib fractures (Figures 2-3). 

The patient was started on antibiotics and steroids for suspected community-acquired pneumonia and COPD exacerbation. Cardiothoracic surgery was consulted for assessment of pulmonary herniation and recommended conservative management. His pain was managed with multimodal therapies including opioids, acetaminophen, lidocaine patch, and topical non-steroidal anti-inflammatory drugs (NSAIDs).

At outpatient follow-up two months following hospital discharge, the patient had good pain control with over-the-counter acetaminophen and topical NSAID creams. His COPD was well-controlled with the use of budesonide inhaler twice daily and as needed albuterol-ipratropium, and he was voluntarily admitted to inpatient rehab to treat his substance use disorder. A palpable bulge was still palpable on exam corresponding to the area of the herniation. A chest plain film was obtained during the visit that showed subsegmental atelectasis of the left lower lung but was otherwise unremarkable.

## Discussion

Lung herniation is an abnormal protrusion of lung parenchyma outside the thoracic cavity. Since the first case report by Roland in 1499 [5], there have been approximately 300 cases reported [1].

Lung herniations are generally classified based on etiology (congenital or acquired) and anatomical location (cervical, thoracic, or diaphragmatic) [6]. Acquired hernias are further subdivided into traumatic, pathologic, or spontaneous. Traumatic lung herniations are the most common type, accounting for 52% of all lung herniations [7]. Common reported causes are uncomplicated rib fractures, motor vehicle accidents, seat belt injuries, penetrating trauma, and blunt force trauma. Traumatic lung herniations may also be related to chest wall surgery or procedures. Interestingly, the herniation can occur years after an initial traumatic event [8].

Pathologic lung herniations are the least common type and have been associated with malignant tumors, abscesses, empyema necessitatis, and tuberculous caries of ribs [8].

Spontaneous lung herniation is a slight misnomer, as it is actually related to chest wall weakness and increased intrathoracic pressure. It may be caused by activities like vigorous coughing, sneezing, heavy lifting, and playing instruments. Smoking, obesity, male sex, COPD, and other pulmonary disease appear to be common features among patients with spontaneous herniations [3].

In our case, history presents both traumatic and spontaneous contributing factors to pulmonary herniation. Our patient’s reported fall may have weakened his chest wall, but he also reported sudden pain associated with vigorous coughing. Furthermore, he had numerous characteristics associated with spontaneous lung herniation, like COPD, chronic cough, and obesity.

Our patient’s cocaine use almost certainly played a role in the development of his lung herniation; this is the first reported case of lung herniation associated with cocaine abuse. Smoking cocaine puts patients at risk for barotrauma, often resulting in acute, vigorous coughing spells [9]. Freebase cocaine users often perform intense Valsalva maneuvers during use to enhance the effects of the drug [10]. The increased intrathoracic pressure from repeated coughing and Valsalva maneuvers could have predisposed our patient to lung herniation and may put him at risk for recurrence in the future.

Given the rare nature of lung herniation, treatment is controversial and based on case reports. The decision between surgical or conservative management depends on the severity of symptoms, hernia size, and comorbid conditions. Early surgical repair has been recommended to prevent complications like incarceration, strangulation, infection, or atelectasis [11].

The conservative approach can involve analgesia, cough suppressants, incentive spirometry, and compression with binders or pads. As in our case, some patients may not tolerate compression due to pain, and binding could result in reduced airflow, predisposing patients to infection and atelectasis. Therefore, analgesia and incentive spirometry are better strategies to promote relaxation of the chest wall muscles and spontaneous reduction [12].

Our patient’s case demonstrates that pain control is a central component of conservative management, as his pain and anxiety limited his ability to take adequate breaths, causing him to require supplemental oxygen. Multimodal analgesia led to improvement of his respiratory status and eventual discharge without need for surgical intervention.

Long-term prognosis for lung herniations is generally good, regardless of management. There are several reported symptomatic cases treated successfully with conservative management, and even reports of spontaneous resolution of the hernia [1,13,14]. In patients who do not have surgery, pain usually improves over time, and progression of the hernia is rare. Recurrence of the hernia is also rare, but a few cases have been reported following both surgery and conservative management. Based on our case and other case reports, conservative management should be considered in a wider range of patients with lung herniations, even those who are symptomatic.

## Conclusions

Lung herniation is a rare cause of acute chest pain. It can be congenital or acquired, most commonly due to trauma or surgery. This pathology can be initially overlooked on chest radiography and CT. Therefore, a high index of suspicion is required both clinically and while reviewing imaging to make an accurate diagnosis. The suspected blunt thoracic trauma secondary to a syncopal event, severe coughing, and cocaine use (due to the associated voluntary increase in intraabdominal pressure to enhance drug effects) were likely all contributing factors for the development of a pulmonary hernia in our patient. Even though standard treatment is not established and based on case reports, a conservative approach is feasible even for symptomatic patients and should have adequate pain control and respiratory hygiene measures as its foundation.
